# Supplementary material for: Venomix: a simple bioinformatic pipeline for identifying and characterizing toxin gene candidates from transcriptomic data
Source: PeerJ. 2018 Jul 31;6:e5361. doi: 10.7717/peerj.5361 (PMC6074769; doi:10.7717/peerj.5361)
Supplement: Supplemental Information 3 [file peerj-06-5361-s003.gz › FinalOutput_E-6/Phospholipase_A1_19/finaltree.pdf]

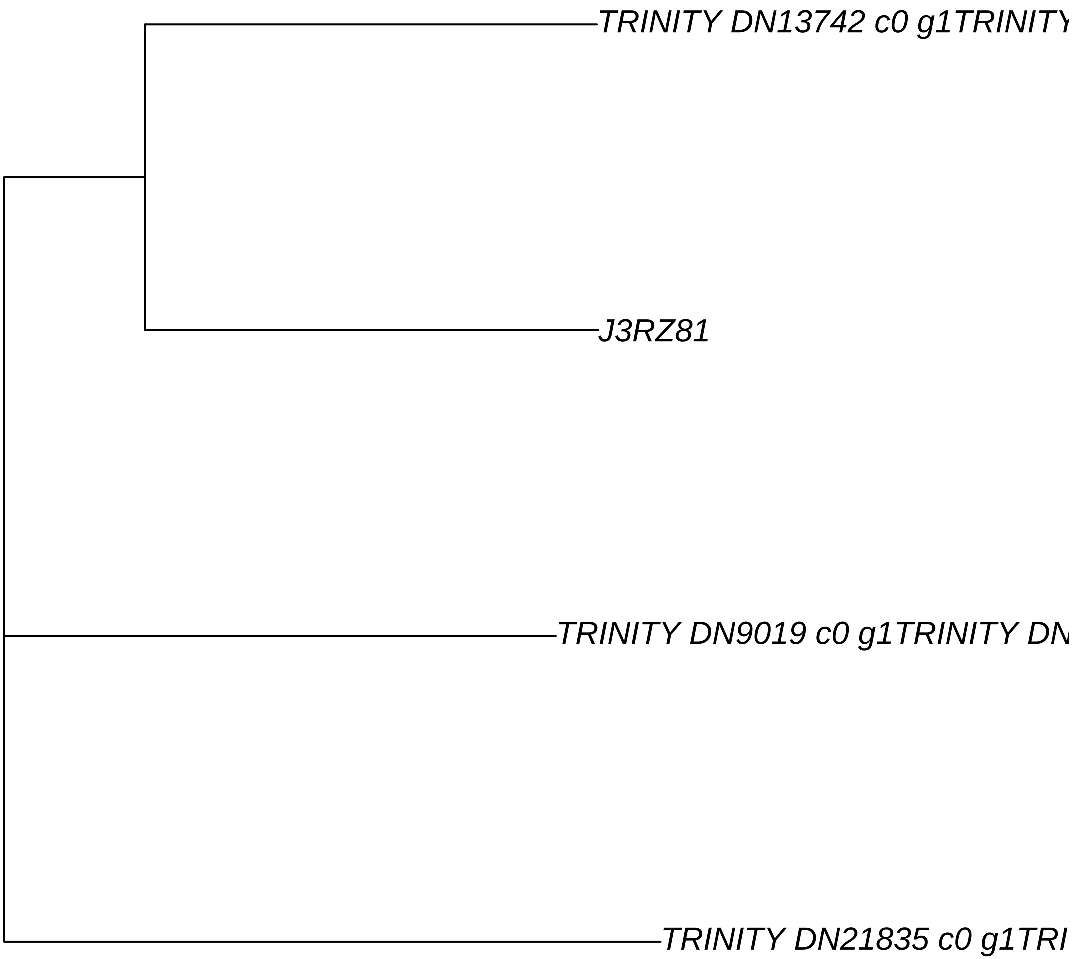
TRINITY DN13742 c0 g1TRINITY
J3RZ81
TRINITY DN9019 c0 g1TRINITY DN
TRINITY DN21835 c0 g1TRI
